# Maternal Effects Underlie Ageing Costs of Growth in the Zebra Finch (*Taeniopygia guttata*)

**DOI:** 10.1371/journal.pone.0097705

**Published:** 2014-05-14

**Authors:** Mathilde L. Tissier, Tony D. Williams, François Criscuolo

**Affiliations:** 1 Département Ecologie, Physiologie et Ethologie, Institut Pluridisciplinaire Hubert Curien, Centre National de la Recherche Scientifique-Université de Strasbourg, Strasbourg, France; 2 Department of Biological Sciences, Simon Fraser University, Burnaby, British Columbia, Canada; UCLA, United States of America

## Abstract

Maternal effects provide a mechanism to adapt offspring phenotype and optimize the mother’s fitness to current environmental conditions. Transferring steroids to the yolk is one way mothers can translate environmental information into potential adaptive signals for offspring. However, maternally-derived hormones might also have adverse effects for offspring. For example, recent data in zebra finch chicks suggested that ageing related-processes (*i.e.* oxidative stress and telomere loss) were increased after egg-injection of corticosterone (CORT). Still, we have few experimental data describing the effect of maternal effects on the growth-ageing trade-off in offspring. Here, we chronically treated pre-laying zebra finch females (*Taeniopygia guttata*) with 17-β-estradiol (E_2_) or CORT, and followed offspring growth and cellular ageing rates (oxidative stress and telomere loss). CORT treatment decreased growth rate in male chicks and increased rate of telomere loss in mothers and female offspring. E_2_ increased body mass gain in male offspring, while reducing oxidative stress in both sexes but without affecting telomere loss. Since shorter telomeres were previously found to be a proxy of individual lifespan in zebra finches, maternal effects may, through pleiotropic effects, be important determinants of offspring life-expectancy by modulating ageing rate during embryo and post-natal growth.

## Introduction

Embryonic and post-natal growth are key phases of the life-cycle, affecting final body size in all organisms and can affect subsequent fitness, *e.g.* through effects on size-dependent fecundity, or reproductive success [1], [2]. Since growth is energetically costly (requiring abundant resources or/and parental care), it is clear that “external” constraints can limit growth [3]. Parental care, predation, social environment, brood size, within-brood size hierarchies, or parasitism can interact and modulate offspring growth [4], [5], [6]. However, “internal” constraints can also limit growth due to the cost of growth itself associated with cell proliferation, cell differentiation and cell growth. Numerous studies looking for a proximate explanation of the decreased lifespan of fast-growing individuals have focused on physiological costs of high growth rates. These studies reveal that accelerated ageing processes can be an internal constraint on fast growth and this might be mediated through a decreased resistance to oxidative stress [7], [8], [9], [10]. Oxidative stress is defined as an imbalance between the production of reactive oxygen species (ROS) and anti-oxidant defenses causing cellular or physiological damage to lipids, proteins or DNA but also including telomere loss [11], [12]. Telomeres are the ends of eukaryotic chromosomes, formed by repeated sequences of non-coding DNA – (TTAGGG)n in vertebrates, protecting the coding part of the chromosome during DNA replication. The role of telomeres in cell senescence and apoptosis is now well established: short and dysfunctional telomeres trigger an emergency cell signaling cascade [13]. Telomeres have more recently been considered at the ‘organismal’ level, first in humans [14] and in free-living animals, where survival rate and lifespan are associated with the longest telomeres and/or the shortest rate of telomere loss in birds [15], [16], [17], [18]. Heidinger *et al.* [18] have highlighted the importance of telomere dynamics during pre- and post-natal growth in terms of future fitness by showing: a) that telomere length and telomere loss in early life can predict the lifespan of individuals in zebra finches (*Taeniopygia guttata*), and b) that relative telomere length at the end of the chick growth phase is preserved throughout adulthood.

Mothers may affect offspring phenotype by modulating physiological costs of growth, notably through hormonally-mediated maternal effects [19]. Maternal transfer of steroid hormones to egg yolk can have significant effects on offspring phenotype, *e.g.* through the down-regulation of offspring growth following CORT treatment [20], [21], [22], a hormone which can inhibit immune responses and standard metabolic rate, and decrease survival rate [6], [23], [24]. Haussmann *et al.* [19] recently showed that embryonic exposure to CORT by egg injection increased oxidative stress and reduced telomere length in domestic chicken (*Gallus domesticus*) chicks when separated from their parents. In contrast, other maternally-derived hormones are known to have a positive impact on ageing mechanisms, *e.g.* E_2_ increases telomerase activity in humans, directly activates a promoter of telomerase [25], and down-regulates oxidative stress by protecting neurons during development [26], reducing ROS production and acting as a potent antioxidant [27], [28] (but see also [29]).

These studies suggest that maternally-derived hormones might have a significant impact on oxidative stress and telomere loss during embryonic and/or post-natal growth to determine individual telomere length at fledging, a fitness-related trait in captive zebra finches [18]. However, the link between hormones, maternal effects, oxidative stress and telomere dynamics remains largely unknown. Here, we investigated the effects of two maternally-derived hormones – CORT and E_2_– on offspring growth and cellular ageing rates (oxidative stress and telomere loss) during early development (*i.e.* embryonic and post-natal growth) in zebra finches. We injected mothers with CORT or E_2_ during egg formation, based on studies showing that an increase in plasma E_2_ or CORT is associated with an increase in maternally-derived yolk steroid hormone levels in eggs produced by these females [20], [23], [30], [31]. We looked at the impact of hormonal treatment on the offspring’s body mass gain (i.e. growth), oxidative stress and telomere dynamics during early development. Due to the potential antagonistic role of these hormones on growth and oxidative stress (see above) we predicted that (1) injections of CORT in mothers would decrease chick growth (body mass gain) and increase oxidative stress and telomere loss during the post-natal growth period, whereas (2) E_2_ injections in mothers would increase growth rates and protect individuals from oxidative stress and telomere loss.

## Materials and Methods

### 1) Animal Care and Breeding Protocol

Zebra finches were maintained in controlled environmental conditions (temperature 19–23°C; humidity 35–55%; constant light schedule, 14L: 10 D, lights on at 07.00). Breeding pairs were provided with a mixed seed diet (*Panicum* and white millet, 1∶3, 11.7% protein, 0.6% lipid and 84.3% carbohydrate by dry mass), water, grit and cuttlefish bone (calcium) *ad libitum*. Breeding pairs were also given a multivitamin supplement in the drinking water once per week and 6 g/pair per day of egg food (eggs, bread crumbs and cornmeal: 20.3% protein, 6.6% lipid) between pairing and fledging. All birds used in this experiment were of the ‘wild type’ plumage morph and were aged 6 months or older. Breeding pairs were placed in separate breeding cages (51 cm×39 cm×43 cm) equipped with an external nest box (14 cm×14.5 cm×20 cm) and nesting material. Nest-boxes were checked daily between 09∶00 and 12∶00 and all new eggs were weighed (to 0.001 g) and numbered. Chicks were raised by their parents until 30 days and then were moved to same-sex cages (46×46×46 cm).

### 2) Ethics Statement

Experiments and animal rearing were conducted under a Simon Fraser University Animal Care Committee permit (901B-94), in accordance with guidelines from the Canadian Committee on Animal Care (CCAC).

### 3) Female Injections and Chick Growth

A total of 45 adult females were weighed (to 0.1 g) and blood sampled (∼ 80 µL from the brachial vein) at the time of pairing, at clutch completion (defined as 2 days after the last egg was laid [32]), and at chick independence (30 days after hatching). Females were randomly assigned to one of the following treatments: CORT, E_2_ or Control (15 females *per* treatment). CORT and E_2_ groups received hormones diluted in canola oil (CORT: 19.2 ng/30 µL of canola oil, E_2_∶16 µg/30 µL of canola oil) while the Control group received only canola oil. Steroids were first dissolved in isopropanol, re-suspended in canola oil and finally the isopropanol was evaporated off under air before the solution was used for injections. Females received intramuscular injections every day between 10∶00 and 11∶00 from day 3 after pairing until clutch completion. Doses were chosen based on previous studies [30], [33] to increase hormones levels in female’s plasma and egg yolks while remaining within physiological levels.

Of the 45 females paired, 30 (9 CORT, 8 Control, 13 E_2_) laid eggs within 2 weeks (66%). Based on the normal distribution of timing of initiation of egg-laying, all the females which hadn’t laid within 2 weeks were removed from the experiment to avoid a bias in the number of injections per female (which varies from 8 to 15 in total, with 1 injection/day). We found no differences between groups in the number on injections received per female (Control: 9.25±1.25; CORT: 9±1.25; E_2_∶10.88±0.83; F_2,14_ = 1.062, p = 0.372). Nest boxes were checked twice a day from day 8 after clutch completion until all the eggs hatched. Hatching occurred in 17 of the 30 nests and the 17 “successful” nests were composed of 4 CORT, 9 E_2_, and 4 Control nests (with a mean number of eggs/female of 4.11, 4.5 and 4.25 for E2, CORT and Control females respectively, F_2;14_ = 0.201 and p = 0.820). The mean hatching success of 56.8% (similar to hatching success reported for some other zebra finch colonies [34], [35]) resulted in a sample size of 15 CORT, 16 E_2_ and 9 Control chicks at hatching and a final sample size of 13 CORT chicks, 8 E_2_ chicks and 9 Control chicks at fledging.

Chicks were marked with a non-toxic pen for individual recognition, weighed to the nearest 0.001 g at hatching and were weighed every two days until 30 days of age (*i.e.* at the end of growth). The sex of chicks which died before sexual maturation was determined using the molecular sexing method adapted from Griffiths *et al*. [36]. Briefly, this method is composed of three steps (i) DNA extraction, (ii) polymerase chain reaction (PCR) and (iii) electrophoresis on an agarose gel to determine the sexual chromosomes of the individuals (ZZ in males and ZW in females). We collected blood samples from nestlings (about 30 µL, from the brachial vein) at 10, 20 and 30 days of age for oxidative stress and telomere measurements. Blood samples were kept on ice and centrifuged at 6000 rpm for 10 minutes within an hour of collection. Chick growth rate was estimated by calculating the mean body mass gain per day during the initial phase of rapid growth (0 to 10 days), and then during the subsequent growth period before separation from the parents (10 to 30 days) in order to see whether hormone treatment differentially affected early and late growth. Chicks undergo rapid, almost linear, growth to 10 days of age whereas the growth period from 10 to 30 days is slower, with chicks starting to feed themselves around 20 days of age [37].

### 4) Telomere Length and Oxidative Stress Assessments

DNA was extracted from 4 µL of red blood cells (nucleated in birds) using DNeasy Blood and Tissue kits (Qiagen, Germany). QIAGEN’s protocol for animal blood (Spin-Column protocol) was followed except that we incubated samples at 56°C for 3 hours in a water bath. Telomere measurements were conducted by quantitative real-time amplification (qPCR) of the telomere sequence, following the procedure described by Criscuolo *et al.* [38] specifically modified for zebra finches (from a protocol initially developed for human samples [39]), widely used since 2009 (*e.g.* [16], [17]). Telomere length measurement is based on the determination of a number of amplification cycles necessary to detect a lower threshold of fluorescent signal. Cycle number is proportional to the sample telomere length (T), or to the number of copies of a non-variable copy number gene (or control gene S). A ratio (T / S) is then calculated for each sample that will reflect relative inter and intra-individual differences in telomere length when repeated samples are taken from the same individual. As a control gene, we used GAPDH (coding for the glyceraldehyde 3-phosphate dehydrogenase), amplified by oligonucleotide primers GAPDH-F (5′-AACCAGCCA-AGTACGATGACAT-3′) and GAPDH-R (5′-CCA-TCAGCAGCAGCCTTCA-3′). For telomeres amplification, primers used were of the following sequences: Tel1b (5′-CGGTTTGTTTGGGTTTGGGTTTGG-GTTTGGGTTTGGGTT-3′) and Tel2b (5′-GGCTTGCCTTACCCTTACCCTTACCCTTAC-CCTTACCCT-3′). Primers – at a concentration of 200 nM/200 nM – were mixed with 5 µL of SYBR Green (a nucleic acid stain), water and 2.5 ng of DNA by well, in a 10 µL final solution. Cycling conditions included an initial hold at 95°C for 2 min for the enzyme activation followed for telomere amplification by 30 cycles of: 1 min denaturation step at 95°C, 30 sec hybridization step at 56°C, and 30 sec elongation step at 72°C including the fluorescence signal readings. A 20 min final melt step was included on each run with the temperature ramping from 56°C to 95°C. The non-VCN gene qPCR conditions were characterized by a 2 min activation step at 95°C followed by 40 cycles of: 1 min 30 sec denaturation step at 95°C, and one step of both hybridization and elongation at 60°C for 1 min, with fluorescence readings recorded at this step. Again, a final melt step ended each run with the temperature ramping from 60°C to 95°C within 20 min. Cycling was performed on a Mastercycler real-time PCR instrument (Eppendorf, Germany). All the samples are run in duplicate and in each run, we included 4 replicates of DNA samples taken from an even number of individuals from each treatment group, as well as a no-template negative control. Mean amplification efficiencies of the qPCR runs were 0.96–0.99 for telomere and between 0.98–1.05 for the non-VCN gene. Intra-plate coefficients of variation (based on CT values) were low: 0.9% for telomere assay and 0.5% for the non-VCN gene assay. Inter-plate coefficients of variation based on repeated samples were 1.9% for the telomere assay and 0.8% for the non-VCN geneassay (based Ct values). Inter-plate coefficients of variation based on the T/S ratio of repeated samples was 4.1%. The melting curves showed a single peak of amplification for samples and no peak for negative control (water).

Oxidative stress was analyzed in plasma samples by measuring ROMs (reactive oxygen metabolites) and OXY (total antioxidant capacity of the plasma) [40]. We used the the d-ROMs test (Diacron International, Grosseto, Italy) and the OXY-ADSORBENT test (Diacron International, Grosseto, Italy), following manufacturer protocols. The d-ROMs test (used on chick’s plasma sample at 10 and 30 days of age) measures the concentration of hydroperoxides, reactive oxygen metabolites produced following an attack of ROS on organic substrates [40]. The OXY-ADSORBENT test (used on chick’s plasma sample at 10 and 30 days of age) measures total antioxidant capacity of plasma (see [41] for details on the procedures). ROMs (*i.e.* damage) were expected to be higher in chicks from CORT-treated females and lower in chicks from E2-treated females, compared to control chicks. Conversely, we were expecting OXY (*i.e.* defenses) to be higher in chicks from E2-treated females in comparison to controls. ROMs and antioxidant concentrations into plasma were assessed by colorimetry (at 510 nm) and expressed in mg of H_2_O_2_/dL and µmol of HClO/mL respectively. Intra-plate variation based on duplicates was below 10% (6.3% and 7.3% for ROMs and OXY respectively) as well as inter-plate variation based on a sample repeated over multiple plates (CV = 5.5% and CV = 6.9% respectively).

### 5) Statistical Analyses

In all our models, hormone treatment, chick sex and treatment*sex interaction were used as fixed factors, and brood size and hatching rank of chicks were included as covariates. We first checked whether (i) egg mass, (ii) hatching body mass and (iii) chick body masses at 10 and 30 days differed among groups, using Linear mixed models (LMM). In these three models, we included (i) mother’s mass, (ii) egg mass and (iii) previous mass as covariates, respectively. Final LMM selection was based on the best AIC (Akaike information criterion) value. Normality was tested by a Kolmogorov-Smirnov test and multiple comparisons were analyzed with a post-hoc LSD (least significant difference) test. All analysis was conducted using SPSS (V.20) and p<0.05 was considered significant.

Then, an independent LMM was used to test whether the changes in body mass during the 30 days of growth differed among hormonal treatments, with the previous mass (*i.e.* the body mass at hatching for the 0 to 10 days period and body mass at 10 days for the 10 to 30 days period) included as a covariate. We controlled for non-independence of individuals in the same nest and the non-independence of repeated measurements on the same individual by including individual identity nested in nest identity as a random effect, time period (0 to 10 days and 0 to 30 days) being the repeated fixed factor in this model. Finally, we conducted LMM analysis for each growth period to test whether hormone treatment affected ROMs and OXY levels (at 10 days first and at 30 days) and whether it affected telomere loss from 10 to 30 days. We included as covariates (i) previous ROMs and OXY levels in the models controlling for oxidative stress levels and (ii) body mass gain, ROMs and OXY at 10 days in the model testing the impact of treatment on telomere loss.

We also used path analysis to test the significance of multiple relationships among observed, potentially inter-related variables and to assess the sign and strength of those relationships [42]. Here, we estimated and quantified hypothesized causal relationships between (i) an exogenous variable: body mass gain (BMG) from 0 to 10 days and four endogenous variables (ii) body mass gain from 10 to 30 days, (iii) ROMs and (iv) OXY at 10 days and (v) telomere loss from 10 to 30 days. Path analyses were conducted independently for each hormone treatment, and selection of the most parsimonious model was made for each treatment by removing the insignificant paths one by one. We ensured that the Chi-square (which tests the null-hypothesis that the reduced model fits the data as well as the saturated model) remained >0.05 when removing a variable. In the control group, BMG from 0 to 10 days and BMG from 0 to 30 days were highly correlated (Spearman correlation, r^2^ = −0.98, p<0.001), potentially generating biased evaluation of the standardized estimates in this group (*i.e.* standardized Betas >1). Based on the paper of Petraitis *et al*. [43], those two highly correlated variables were merged in one variable (BMG from 0 to 30 days) in order to avoid a bias caused by a problem of co-linearity in the path analysis.

## Results

### 1) Oxidative Stress and Telomere Length of Mothers

ROMs levels and relative telomere loss of mothers were affected by our experimental treatment (F_2,6_ = 6.872, p = 0.028 and *F_2,7_ = 4.723, p = 0.050*). Post-hoc analyses indicated that mothers injected with CORT had more ROMs than Control mothers (11.927±1.543 mg H_2_O_2_/dL and 6.067±1.579 mg H_2_O_2_/dL respectively, p = 0.010) when their chicks were 30 days old, relative to their levels at pairing. CORT-treated mothers also had a higher rate of telomere loss than Control mothers (−0.631±0.138 vs. −0.032±0.139, p = 0.018). However, we found no significant differences in those parameters between E_2_-treated females and either CORT- or control mothers. OXY levels and DNA damage of mothers were not affected by our hormonal treatments (F_2,6_ = 0.657, p = 0.552, F_2,4_ = 0.575, p = 0.603).

### 2) Treatment Effects on Embryonic and Post-natal Growth

Egg mass differed significantly among treatments (F_2,154_ = 6.873, p = 0.001): eggs produced by E_2_-treated females were significantly heavier (1.101±0.055 g) than eggs produced by Control (1.047±0.056 g) and CORT-treated females (1.044±0.056 g, p = 0.003 and p = 0.002 respectively). Similarly, hatching body mass of chicks was significantly affected by mother’s hormonal treatment (F_2,30_ = 5.918, p = 0.007). However, here chicks of CORT-treated females were lighter (0.635±0.062 g) than chicks of Control- (0.783±0.066 g) or E_2-_treated females (0.767±0.065 g, p = 0.002 and p = 0.017 respectively).

**Figure 1:**
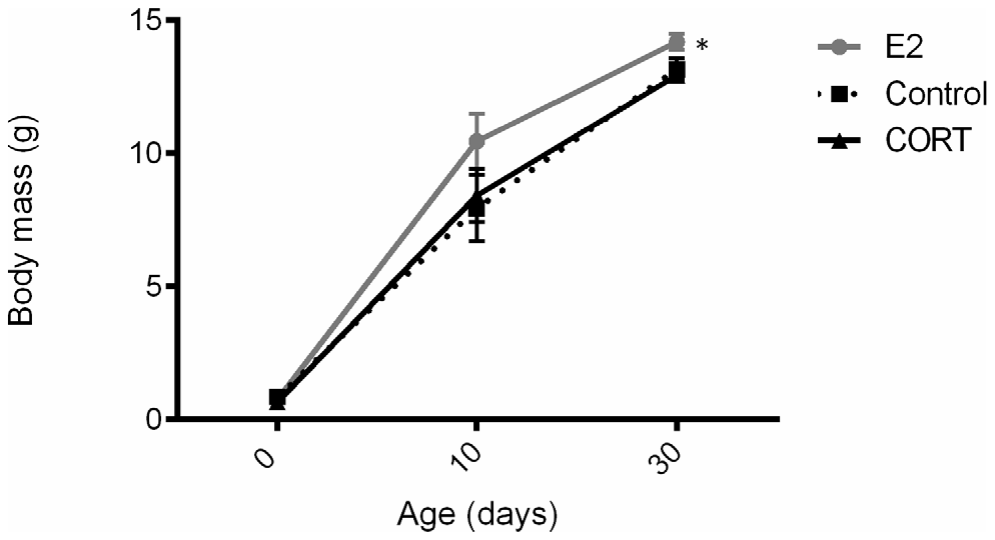
Body mass dynamics of male chicks raised by hormonally-treated (E_2_ and CORT) and control zebra finch females. This higher body mass in E2 male chicks results from a significantly higher body mass gain over the first 30(see text for details). Chicks were measured at hatching and every two days until 30 days of age. Grey dots, black squares and black triangles represent E_2_, Control and CORT treated chicks, respectively. Means are plotted ± SE and * indicates a significant difference in body mass.

There was a significant sex*treatment interaction (F_2,29.671_ = 4.276, p = 0.023) for body mass gain of chicks. We therefore conducted a separate analysis of treatment effects by chick sex which showed that hormone treatment modified growth rate in male chicks (F_2,10.560_ = 4.276, p = 0.008) but not in female chicks (F_2,11.510_ = 0.063, p = 0.939). Post-hoc analyses revealed that male chicks of E2-treated mothers had higher growth rates than male chicks of CORT-treated females (Figure 1, p = 0.002). However, growth rate of E_2_ male chicks was marginally non-significantly different compared with Control male chicks (Figure 1, p = 0.063). This sex-specific effect was confirmed for body mass of chicks at 30 days of age (F_2,22_ = 4.156 et p = 0.029): male chicks of E_2_-treated females were heavier than Control and CORT males (Figure 1, p = 0.030 and p = 0.009 respectively).

### 3) Treatment Effects on Chick Oxidative Stress and Telomere Dynamics

**Figure 2:**
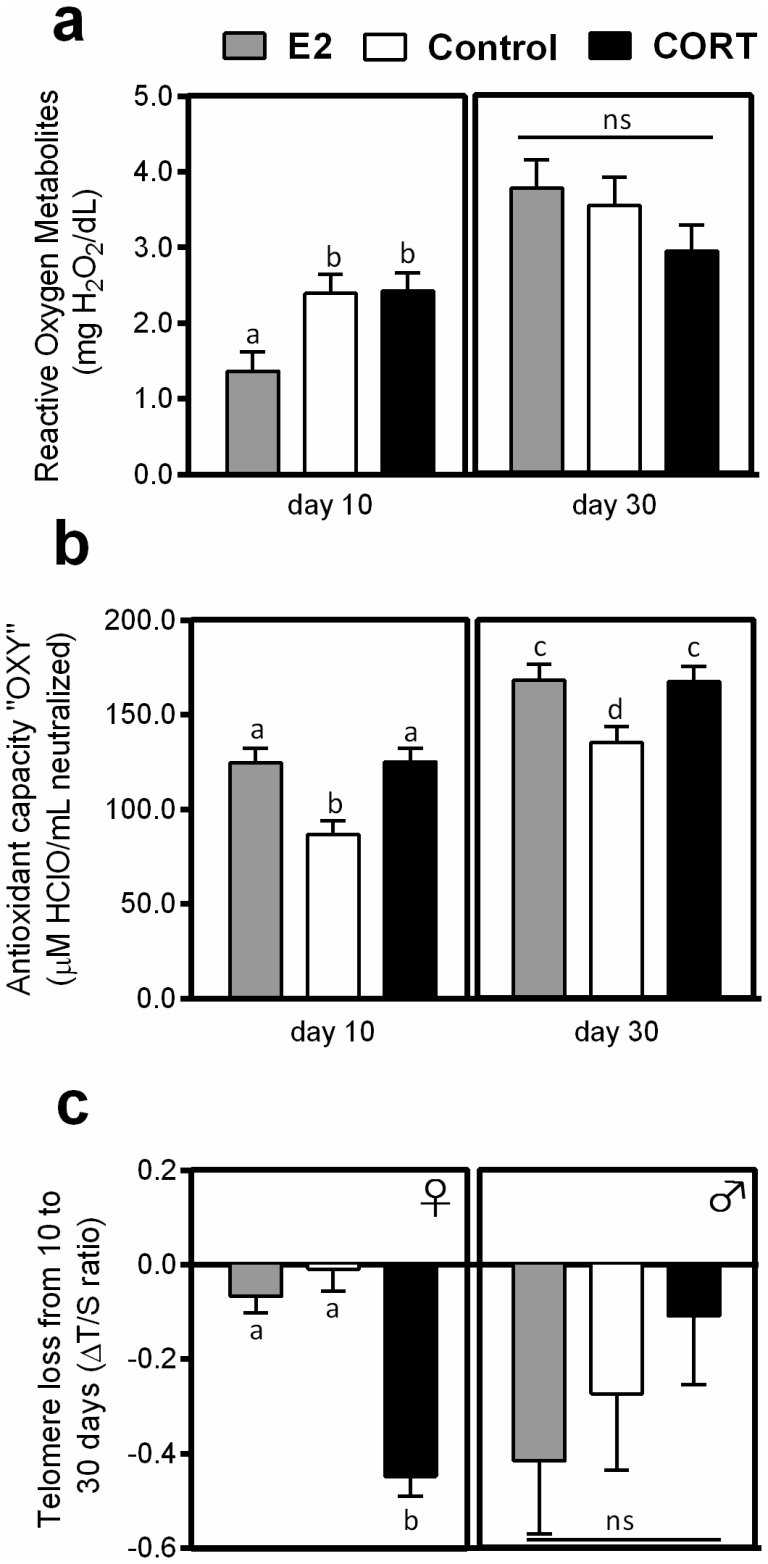
Effect of the maternal hormonal treatment on ageing markers of chicks from E_2_-treated females (grey), control females (white) and CORT-treated females (black) zebra finch. Plasma reactive oxygen metabolites (a), antioxidant capacity (b) and red blood cell telomere loss (c) of chicks are indicated (N = 29∶8 E2, 8 Control and 13 CORT chicks; Means ± SE). Different letters highlight significant inter-group differences (p<0.05). Proper statistical analysis is detailed in the text.

Hormone treatment of mothers had a significant effect on ROMs levels of 10 days-old chicks (F_2,23_ = 5.012, p = 0.016). E_2_ chicks from both sexes had lower ROMs levels than Control and CORT chicks (Figure 2a, p = 0.013 and p = 0.008 respectively). Chick ROMs levels also differed between sexes at 10 days (F_1,23_ = 5.872, p = 0.024): male chicks had lower ROMs levels than females (1.732±0.465 mg H_2_O_2_/dL and 2.398±0.461 mg H_2_O_2_/dL respectively). However, there was no significant effect of treatment or sex on ROMs at 30 days (Figure 2a, F_2,20.863_ = 1.586, p = 0.228). Treating mothers with CORT and E_2_ also had a significant impact on plasma antioxidant capacity of their chicks at 10 days (F_2,21_ = 8.747 and p = 0.002). E_2_ and CORT chicks had a higher antioxidant capacity than Control chicks (Figure 2b, p = 0.006 and p = 0.001 respectively). The same difference was observed at 30 days (F_2,22.949_ = 7.617, p = 0.003): Control chicks had reduced antioxidant capacity compared to E_2_ and CORT chicks (Figure 2b, p = 0.003 and p = 0.002 respectively).

Hormone treatment also had a significant effect on telomere loss of chicks between 10 and 30 days of age (Figure 2c): treatment*sex interaction (F_2,20_ = 3.227, p = 0.048). While no significant treatment effect was found in male chicks (F_2,5.26_ = 0.871, p = 0.471), there was a significant effect of hormone treatment in female chicks (F_2,9_ = 6.652, p = 0.017). Post-hoc tests indicated that female chicks from CORT-treated mothers had greater telomere loss compared to Control and E_2_ chicks (Figure 2c., p = 0.009 and p = 0.025 respectively).

### 4) Path Analysis and Correlated Relationships Between

#### Body mass gain and ageing variables

**Figure 3:**
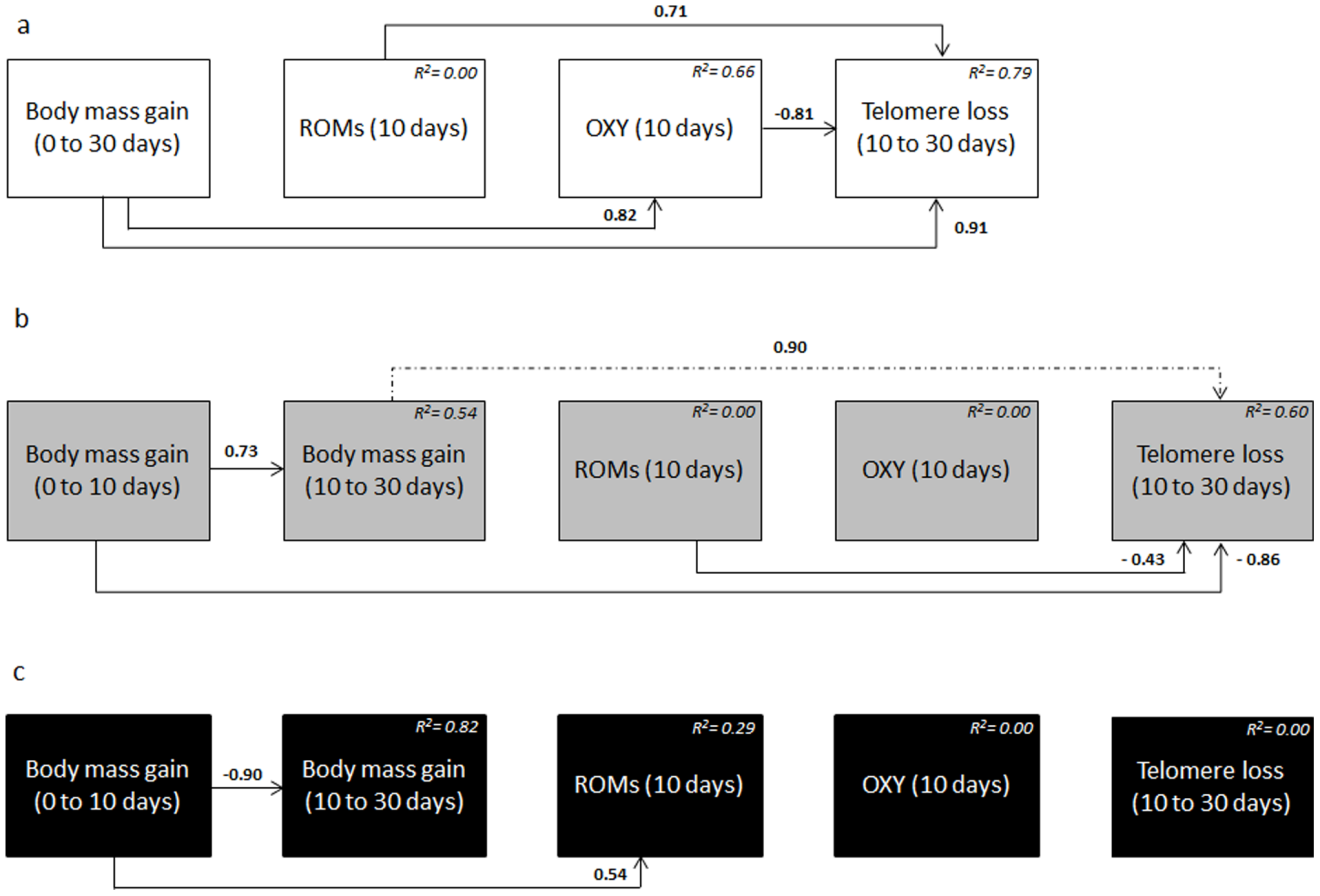
Path analysis diagrams for intra-group relationships between growth and ageing variables measured on chicks, in response to the hormonal treatment of the reproductive mothers. Control (a), E_2_ (b) and CORT group (c). Arrow represent causal link between variables, and standardized estimates (which can be positive or negative) are indicated along their respective paths. The total effect is the sum of direct and indirect effects. Significant paths are indicated by solid arrows while the dashed arrow represents a non-significant path remaining in the selected model. R^2^ is indicated in the box of each exogenous variable and represents the variation of the endogenous variable (*i.e.* body mass gain) explained by each corresponding exogenous variables. See text for statistical details. **ROMs:** reactive oxygen metabolites; **OXY:** plasma antioxidant capacities.

Multiple regression analysis (conducted for each treatment separately) show that body mass gain from 0 to 30 days was positively correlated with OXY levels at 10 days in Control chicks (Figure 3a, p<0.001). In CORT chicks, body mass gain from 0 to 10 days was significantly and positively correlated with ROMs levels in (Figure 3c, p<0.001 and p = 0.025). However, no correlations were found between body mass gain and OXY or ROMs levels in E_2_ chicks (Figure 3b, p>0.05). Body mass gain from 0 to 10 days also contributed directly but negatively to the rate of body mass gain recorded between 10 to 30 days in CORT chicks (Figure 3c, p<0.001) but positively in the E_2_ group (Figure 3b, p = 0.005). In other words, E_2_-treated chicks with the fastest growth between 0 and 10 days also had the fastest growth between 10 and 30 days but without any associated increase in their plasma d-ROMs levels or any decrease in their plasma OXY levels. Finally, body mass gain contributed directly to telomere loss between 10 and 30 days in two cases: (i) positively in Control chicks (Figure 3a, p = 0.003), with faster growing chicks having higher telomere loss, and (ii) negatively in E_2_ chicks (Figure 3b, p = 0.011). This suggests, counter-intuitively, that the chicks raised by E_2_-treated females that grew faster from 0 to 10 days had reduced telomere loss subsequently. No significant interactions were observed in the CORT chicks between these two variables.

#### Oxidative stress and telomere loss

ROMs levels at 10 days contributed to the rate of telomere loss between 10 and 30 days in Control chicks (positively, Figure 3a, p<0.001) and E_2_-treated chicks (negatively, Figure 3b, p = 0.05). No significant relationships between these variables were found in CORT chicks (Figure 3c, p>0.05). In the control group, there was also a significant and negative contribution of antioxidant levels at 10 days on subsequent telomere loss (Figure 3a, p = 0.007).

## Discussion

Our study design examined the effect of an experimental manipulation of the maternal hormonal state during egg formation and egg-laying on chick growth rate, assuming transfer of steroids from mothers to yolks. We subsequently studied the direct or indirect consequences of this hormonal treatment on chick ageing processes – *i.e.* oxidative stress and telomere dynamics – during embryonic and post-hatching growth. Our study therefore only focused on the global impact of maternal hormonal status on chick phenotype, and does not distinguish between either pre- or post-natal effects or between direct (hormonal) or indirect (parental care) mediated impacts of our maternal treatment. Using two steroid hormones that are thought to have pro- (CORT) or anti-ageing effects (E_2_), we showed that maternal exposure to CORT lead to a reduced hatching body mass in chicks of both sexes, and to an increased rate of telomere loss in female chicks. Conversely, maternal exposure to E_2_ increased body mass gain and body mass at the end of the growth period (30 days of age) in male chicks, but this was not associated with increased oxidative stress or telomere loss as usually observed in young with increased growth rates [8], [10], [28], [55]. Path analyses revealed complex modulation of the relationships between the inter-related variables of growth, oxidative stress and telomere dynamics in chicks reared by CORT and E2-treated females which suggest that the whole balance between pro- and anti-telomere erosion processes was likely affected by our experimental treatment.

### 1) Maternal Hormonal Treatment

Our experimental protocol involved hormonal treatment of mothers assuming transfer of steroids from mothers to yolks (following previous studies [20], [23], [30], [31]), rather than direct manipulation of egg hormone content by egg injection. This raises the possibility that the effects we observed on offspring could have been ‘indirect’, *e.g.* mediated by modifications of egg composition or post-hatching changes in parental care, rather than ‘direct’ effects of maternally-derived hormones. For example, Christians & Williams [44] reported that E2 treatment had no effect on egg mass or egg albumen content in European starlings but eggs of E2-treated females had less yolk lipid (−12%) and yolk protein (−12%), However, in our study egg mass and chick mass were actually *higher* following E2 injections in females. Salvante & Williams [33] showed that CORT treatment had no effect on egg mass, yolk protein, total protein, yolk lipid, or dry masses of egg shell, albumen, or yolk in zebra finches, despite experimental females having elevated plasma CORT during egg-laying. However, we cannot rule out the possibility that hormone treatment affected other aspects of egg quality (*e.g.* antioxidant, immunoglobulin levels) that contributed to differences in chick oxidative state. It is also possible that our hormonal treatment may have affected chick traits via some post-hatching negative effect on female condition or quality or parental care, although Salvante & Williams [33] showed that CORT treatment had no effect on the pattern of change in female body mass during egg-laying. Similarly, although we have no detailed data on parental care, hormone-treated females were still able to contribute to successfully to hatching and rearing chicks in our study. We know from previous studies that hormones injected into mothers are transferred to eggs [20], [23], [30], [31], resulting in elevated yolk steroid levels. In addition, at least for CORT, when injected into eggs, hormones do have direct effects on chicks’ phenotypes similar to those we document here [45], [46]. Additional measurements of hormone levels in egg yolk in our case would help to better understand the precise mechanism involved in the plasticity of chick’s growth and ageing phenotype that we document.

### 2) Maternal Hormonal Treatment and Offspring Growth

Chicks of CORT-treated females had a reduced body mass at hatching compared to chicks of control and E2 groups suggesting that CORT injections in mothers lead to a reduced growth during pre-natal development. This association between CORT and growth reduction has been well documented [20], [21], [47]. However, this impact seems to diminish during post-hatching development in our study: CORT-treated male chicks did not show significantly reduced growth or body masses at 30 days compared to controls. This is consistent with other studies that found no effect of CORT on growth or fledging body mass [19], [23]. Conversely, male chicks from E2-treated females had higher growth than CORT males (marginally non-significantly different than Control males) and a higher mass at 30 days. These results suggest that E2 injections promoted growth of male chicks during post-natal development, while CORT injections seem to have acted mainly during embryo development. Our treatment could have affected offspring growth in different ways. First, the treatment could have indirectly affected chick growth by modulating maternal behavior and investment in offspring. Indeed, the increased egg mass of E_2_-injected females in our study suggests an increase in reproductive investment to embryos, as previously noted by Williams [48]. This higher investment in reproduction should then lead to a better fitness for the mother, through “better quality” offspring, which is consistent to our finding of increased body mass growth and increased 30 days-old body masses of E_2_ compared to CORT offspring males. Alternatively, our results could be due to a direct modulation of chick growth by maternally-derived E2 and CORT. Unfortunately, to our knowledge, studies connecting E_2_ and growth are lacking, particularly in birds (but see [49], [50]). Interestingly, the impact of CORT injections on embryonic development is better documented [6], [19], [20], [33], [47]. Hormone treatment also affected ageing parameters of mothers, with CORT negatively affecting oxidative stress and telomere maintenance. Indeed, CORT-treated females had higher levels of ROMs and shorter telomeres at fledging, suggesting an ageing cost of CORT injections [19]. These findings, associated with the increased egg mass of E_2_-treated females, suggest that our hormonal treatment was physiologically relevant.

### 3) Maternal Hormonal Treatment and Offspring Ageing Parameters

E_2_ injections could also have had an effect on chick growth through the down regulation of oxidative stress (lower levels of oxidative damages and higher levels of plasma antioxidant capacity). Indeed, as discussed above, oxidative stress has been suggested to have modulatory effects on growth rate [12]. Interestingly, E_2_ males grew faster but did not suffer increased oxidative damage which is consistent with estrogen’s capacity to reduce ROS production [27] (reviewed in [28]; but see also [51]) or its ability to be a potent antioxidant [28]. Contrary to our predictions – and unlike a previous study [19] – we found no impact of CORT on oxidative damage (*i.e.* ROMs levels and DNA damage) either at 10 or 30 days, perhaps because CORT chicks had higher levels of plasma antioxidant capacities (*i.e.* OXY) in our study. However, our results are consistent with the finding of Hoogenboom *et al.* [52] in brown trout, where cortisol levels were not directly related to oxidative damage. The effects of CORT on physiology and behaviour have been shown to vary widely according to dose, timing, species and life-history strategy [53], [54], [55]. This could partly explain these contrasting findings of studies on CORT impacts on oxidative balance.

Even though we found no effects of CORT-treatment on chick’s oxidative damage, and no correlation between ROMs levels and telomere loss (figure 3c), female chicks of CORT-treated mothers had greater telomere loss between 10 and 30 days than female chicks of the other groups. This telomere loss was not related to rate of body mass gain, ROMs levels or OXY levels (figure 3c) contrary to what we observed in the Control chicks (figure 3a). Under enhanced CORT-levels, telomere erosion due to cell division might have been costly because of the inhibition of telomere maintenance process by CORT, *i.e.* the inhibitory effect of CORT on telomerase [56]. On the other hand, there was no effect of hormone treatment, or of increased growth rate, on telomere loss in E_2_ chicks. As shown in figure 3b, the link between oxidative damage (ROMs) and telomere loss, and the link between body mass gain from and telomere loss, are the reverse of the relationships observed in Control chicks (figure 3a.). This suggests that the relationships between growth, oxidative stress and telomere loss have been changed by E_2_ treatment, hypothetically through a positive impact of E_2_ on telomerase activity. Indeed, besides its antioxidant capacities, estrogen is also known to be an activator of telomerase [25] and to reduce telomere loss in humans [27].

### 4) Maternal Hormonal Treatment and Ageing Rate Consequences for Offspring

Our data suggest that E_2_ treatment but not CORT treatment, has a sex-specific effect on post-natal growth in offspring with no effect on embryonic development. This effect of E2 is associated with short-term physiological benefits (*i.e*. decreased oxidative damage and increased plasma antioxidant capacities), and no apparent costs in terms of increased telomere loss, perhaps due to a positive effect of E_2_ on telomerase activity. On the other hand, maternally derived CORT seems to have an impact on offspring embryonic development, in both males and females, and a sex-biased long term cost on cellular ageing processes, affecting only telomere loss in female chicks in zebra finches, again potentially because of decreased activity of telomerase in females. Sex-specific effects of hormonal treatment on growth and on offspring phenotype have previously been revealed in birds [21], [57], [58]. They could be explained by indirect differential investment by mothers into male and female offspring, triggered by E_2_ or CORT treatment in our experiment, or by different direct sex-specific effects of these hormones. An experiment utilizing egg injection, directly manipulating hormone exposure of embryos, would allow us to discriminate between maternal-derived and direct effects of hormones on chicks’ phenotypes and to understand if maternal hormones act directly on ageing parameters. Furthermore, Haussmann *et al.* [19] showed that CORT injection in eggs influence chick ageing in chicken and here we show that maternal-hormonal status can also modulate growth and ageing in chicks. The next step would be to test whether natural variability in maternal E_2_ and CORT levels is of importance in determining chick phenotype at fledging.

It remains important to evaluate the consequences of these effects on future offspring fitness. Based on the recent literature, shortened telomere length at the end of the growth period is deleterious in terms of lifespan in captive zebra finches [18]. A comparable conclusion was found in mammals, for which poor nutritional conditions in early life resulted in shortened telomeres and reduced lifespan [59], [60]. Given that measuring telomere length in blood cells partially reflects telomere dynamics in other tissues [61], [62], [63], this suggests that having long telomeres is beneficial (see also [64], [65] and references herein). As such, in our case this suggests that CORT- and E2-treated offspring might have contrasting life-history trajectories. A similar hypothetical conclusion can be drawn from our demonstration of a lack of cost in terms of oxidative damage despite a faster growth rate in E2 offspring. Given that oxidative damage has been proposed to mediate longevity costs of faster growth (reviewed in Monaghan *et al.* [12]), and that high oxidative damage levels have been related to reduced fertility or survival rate in natural conditions (*e.g.* Bize et al. [16]), E2 offspring may also beneficiate from fitness advantages. Testing both the accuracy of these expected long-term effects of our experimental maternal-treatment and the pathways actually involved (direct hormonal modulation of the offspring phenotype or through parental care mediated effects) will be a future crucial step to understand the evolutionary value of maternal effects mediated by CORT and E2.
